# Supplementary material for: The Rationale and Design of the KOSovan Acute Coronary Syndrome (KOS-ACS) Registry
Source: Diagnostics (Basel). 2024 Jul 11;14(14):1486. doi: 10.3390/diagnostics14141486 (PMC11276365; doi:10.3390/diagnostics14141486)
Supplement: Supplementary file 1 [file diagnostics-14-01486-s001.zip › diagnostics-2895764-supplementary.pdf]

Supplementary Data

I. List of the data that will be collected

1. Data for the medical center/hospital where patients with Acute Coronary Syndrome will be diagnosed, admitted and managed:
   - Type of hospital (private or public).
   - Number of beds in the hospital.
   - Whether it has Cath lab, PCI, and Cardiac Surgery on site.
   - Total number of MI cases, PCIs and primary PCIs by the center per year.
   - The type of usual treatment for STEMI patients (Primary PCI or Thrombolysis).

2. Informed Consent signed by the patient or patient's family, and information whether the patient is a STEMI patient, and above 18 years of age.

3. Patient characteristics
   - Basic information: gender, date of birth, height, weight.
   - Patient's clinical history: previous MI, angina, PCI, CABG, and Stroke/TIA; presence of Chronic heart failure (and NYHA class), diabetes mellitus, hypercholesterolemia, atrial fibrillation, peripheral vascular disease, current malignant disease, sleep apnea, hypertension, smoking status, history of dialysis treatment, and any other life-limiting disease.

4. Admission process
   - Date and time of: symptoms onset, call for medical help, and first medical contact.
   - Type of the first medical contact: general practitioner, paramedical or medical ambulance, emergency room staff, or other.
   - Admission mode (via ambulance or self-presented) and site (whether the patient is admitted directly to a PCI center or in hospital that does not provide PCI).
   - Date and time of first qualifying ECG, and of the arrival at the non-PCI and/or PCI hospital.
   - Information if the patient had out of hospital cardiac arrest.

5. Presentation and Initial assessment

- ECG data: heart rate, type of STEMI (anterior or other), LBBB, Atrial fibrillation, and pacemaker rhythm.
- Systolic blood pressure at presentation, Killip class, ventilation status (if yes: before or during admission), and information whether the patient is undergoing therapeutic hypothermia.

6. Treatments
   - Intended treatments for the STEMI: primary PCI at the actual center, referral to another PCI center, thrombolysis, or no reperfusion (information about the reason of no reperfusion: whether it's clinically inappropriate, there are contraindications to pharmacological treatments, late presentation, spontaneous reperfusion, wrong diagnosis, patient refusal, or other reasons to be specified).
   - Treatment given for the STEMI case: If it's a primary PCI, date and time will be specified. If it's thrombolysis, date time and place (ambulance, ER, CCU/ICU) will be specified. If PCI was planned but cancelled following the angiography, the reason should be noted in the form.
   - Details of coronary anatomy and PCI procedure: Data collected regarding coronary anatomy and PCI procedure will include information on the type of the procedure, whether is primary or not, the arterial access (femoral, radial, or other), LMS stenosis (if it's more than 50% or not), the number of epicardial territories with stenoses of more than 50%, the identification and the specifying of the Culprit vessel, TIMI flow pre and post procedure, data on thrombectomy (if used during the procedure), the type of stent used, and if non culprit lesions are treated during the procedure or not. Also, data collection includes information on hemodynamic support and the type, when used.

7. Data collected during hospitalization until discharge will include:
   - Last measured ejection fraction, earliest and lowest hemoglobin level, earliest creatinine level, LDL cholesterol, total cholesterol, and glucose plasma levels.
   - Bleeding events, information regarding blood transfusions, and if the patient suffers a cardiovascular event, the type should be specified.
   - Data on staged PCI (whether performed during the index hospitalization, planned for another admission, or not performed at all), CABG (emergent or not-emergent), re-infarction, stent thrombosis, and mechanical complications and their type (tamponade, VSD, or none). Information on heart failure and worst Killip class during hospitalization will also be collected.

8. Data collected at discharge will include:

- Date of discharge and destination (home, nursing home, or another hospital), final diagnosis, and planned rehabilitation.
- If the patient dies during hospitalization, the data collected should include date and time of death, and the cause of it.

9. Medications

   Data collection will include medications used chronically before the event, the prehospital treatment, the treatment during the first 24 hours after admission, and the treatment at discharge, for the following:
   - Antiplatelets (the exact drugs should be specified), Anticoagulants (the exact drugs should be specified), Beta-blockers, ACE inhibitors, ARBs, MRAs, Digoxin, Diuretics, Ivabradine, Statins (the exact drugs should be specified), other lipid-lowering agents (the exact drugs should be specified), PPIs, and other drugs.

10. Follow up

During the one-year follow-up, the following data will be collected:

- The date of the follow-up, and how the follow-up is performed (telephone call or clinical visit).
- If the patient died during the follow-up period, the date and cause of death should be specified. If alive, NYHA and CCS classes should be recorded.
- Major clinical events such as myocardial infarction, unstable angina, stent thrombosis, stroke, and heart failure events, and hospitalization dates for each of these events, if any.
- Any clinical procedure (and the date when performed) during the follow-up period, including angiography, PCI, CABG, ICD-implantation, and resynchronization therapy.
- Laboratory and other examinations such as: heart rate, blood pressure, ejection fraction, creatinine, and LDL cholesterol.
- Medications used after discharge including: Antiplatelets (the exact drugs should be specified), Anticoagulants (the exact drugs should be specified), Beta-blockers, ACE inhibitors, ARBs, MRAs, Statins (the exact drugs should be specified), and other lipid lowering agents (the exact drugs should be specified).

I. List of participating centers:

1. University Clinical Centre of Kosova, Prishtina, Kosovo
2. Regional Hospital of Mitrovica, Mitrovica, Kosovo
3. Regional Hospital of Peja, Peja, Kosovo
4. Regional Hospital of Prizren, Prizren, Kosovo
5. Regional Hospital of Gjakova, Gjakova, Kosovo
6. Regional Hospital of Gjilan, Gjilan, Kosovo
7. Regional Hospital of Ferizaj, Ferizaj, Kosovo
8. American Hospital, Prishtina, Kosovo
9. United Hospital, Prishtina, Kosovo
10. Premium Hospital, Prishtina, Kosovo
11. Aloka Hospital, Prishtina, Kosovo
